# Tetrahydrobiopterin Supplementation Improves Endothelial Function But Does Not Alter Aortic Stiffness in Patients With Rheumatoid Arthritis

**DOI:** 10.1161/JAHA.115.002762

**Published:** 2016-02-19

**Authors:** Kaisa M. Mäki‐Petäjä, Lisa Day, Joseph Cheriyan, Frances C. Hall, Andrew J. K. Östör, Nicholas Shenker, Ian B. Wilkinson

**Affiliations:** ^1^ Division of Experimental Medicine and ImmunotherapeuticsAddenbrooke's HospitalUniversity of CambridgeUK; ^2^ Rheumatology Research UnitAddenbrooke's HospitalUniversity of CambridgeUK

**Keywords:** arteriosclerosis, endothelial function, inflammation, rheumatoid arthritis, tetrahydrobiopterin, Endothelium/Vascular Type/Nitric Oxide, Inflammation, Oxidant Stress

## Abstract

**Background:**

Rheumatoid arthritis is a systemic inflammatory condition associated with increased cardiovascular risk that may be due to underlying endothelial dysfunction and subsequent aortic stiffening. We hypothesized that supplementation with tetrahydrobiopterin (BH_4_) would recouple endothelial nitric oxide synthase and thus improve endothelial function and consequently reduce aortic stiffness.

**Methods and Results:**

We conducted 2 randomized, double‐blinded, placebo‐controlled crossover studies examining 2 separate regimens: an acute regimen, with a single dose of BH_4_ 400 mg versus placebo (n=18), and a short‐term regimen, composed of a 1‐week treatment with BH_4_ 400 mg once daily versus placebo (n=15). Flow‐mediated dilatation and aortic pulse wave velocity were studied 4 times, before and after each treatment phase. Acute BH_4_ supplementation led to an improvement of flow‐mediated dilatation, whereas placebo had no effect (mean±SD of effect difference 2.56±4.79%; *P*=0.03). Similarly, 1‐week treatment with BH_4_ improved endothelial function, but there was no change with placebo (mean±SD of effect difference 3.50±5.05%; *P*=0.02). There was no change in aortic pulse wave velocity following acute or short‐term BH_4_ supplementation or placebo (mean±SD of effect difference: acute 0.09±0.67 m/s, *P*=0.6; short‐term 0.03±1.46 m/s, *P*=0.9).

**Conclusion:**

Both acute and short‐term supplementation with oral BH_4_ improved endothelial function but not aortic stiffness. This result suggests that BH_4_ supplementation may be beneficial for patients with rheumatoid arthritis by improving endothelial dysfunction and potentially reducing risk of cardiovascular disease. There appears to be no causal relationship between endothelial function and aortic stiffness, suggesting that they occur in parallel, although they may share common risk factors such as inflammation.

## Introduction

Rheumatoid arthritis (RA) and other chronic inflammatory diseases are associated with increased premature mortality, mostly due to an excess of cardiovascular disease (CVD).^1^, ^2^ A number of underlying mechanisms have been proposed, including endothelial dysfunction. Reduced nitric oxide (NO) bioavailability or endothelial dysfunction is thought to be a key early step in the initiation of atherosclerosis and predicts future CVD‐related events in a variety of populations, improving risk classification beyond the Framingham Heart Study scores.^3^, ^4^ We and others have demonstrated that RA patients have endothelial dysfunction,^5^, ^6^, ^7^, ^8^, ^9^ which can be improved with anti‐inflammatory therapies.^10^, ^11^, ^12^ Aortic stiffening, an independent risk factor for CVD,^13^ has also been implicated. Several groups have reported increased aortic pulse wave velocity (aPWV) in RA and suggested that this could be a consequence of endothelial dysfunction.^10^, ^14^, ^15^, ^16^ Previous studies suggested a link between endothelial function and stiffness of muscular arteries,^17^, ^18^ but whether endothelial function is causally related to aortic stiffness is unknown.

The mechanism of endothelial dysfunction in RA is incompletely understood, but reduced tetrahydrobiopterin (BH_4_) availability or stability^19^ and increased production of reactive oxygen species have been suggested.^20^ Under basal conditions, endothelial nitric oxide synthase (eNOS) is responsible for NO production, but during inflammation, inducible NOS is also expressed in arterial endothelial cells. Inducible NOS activation impairs vasorelaxation in vitro, at least in part, by limiting availability of BH_4_ to eNOS,^21^ and we have demonstrated in vivo that inducible NOS activation in RA is independently related to endothelial function.^9^ BH_4_ deficiency is thought to lead to eNOS uncoupling, in which electrons flowing from the reductase domain to the oxygenase domain are diverted to molecular oxygen rather than l‐arginine, leading to production of superoxide (O_2_^−^) rather than NO.^22^ Increased release of myeloperoxidase from activated neutrophils may also play an important role in endothelial dysfunction in RA. Myeloperoxidase catalytically consumes NO and increases the production of reactive oxygen species, subsequently leading to uncoupling of eNOS^20^ and oxidation of BH_4_ to inactive BH_2._ 19, 23, 24 We and others have shown that circulating myeloperoxidase levels are elevated in patients with RA^9^, ^25^ and independently predict endothelial dysfunction in patients with unstable coronary disease^26^ and RA.^9^ Inflammation appears to reduce BH_4_ bioavailability, which may in part mediate the endothelial dysfunction present in RA; therefore, supplementation with BH_4_ may be a reasonable therapeutic option to restore normal eNOS function and thus endothelial function in RA. Previous studies have demonstrated that intra‐arterial administration of BH_4_ improves endothelium‐dependent dilation in patients with hypercholesterolemia,^27^ coronary artery disease,^28^ and type II diabetes.^29^ Oral BH_4_ supplementation improves endothelial function in elderly participants,^30^ in long‐term smokers,^31^ and in patients with hypercholesterolemia^32^ and hypertension.^33^ No studies to date, however, have investigated the effects of BH_4_ in patients with RA. Moreover, it is unclear if improvement of endothelial function would lead to a reduction of aortic stiffness or if other mechanisms are responsible for increased aortic stiffness.

Our aim was to test the hypotheses (1) that oral BH_4_ supplementation would improve endothelial function in patients with RA and (2) that an improvement of endothelial function would lead to subsequent amelioration of aortic stiffening, providing evidence about the causal relationship between endothelial function and aortic stiffness; BH_4_ is thought to have little direct effect on inflammation per se.

## Methods

### Study Population

Overall, 33 patients with active RA (disease activity score [DAS28] >3.5) who met the 1987 American Rheumatism Association criteria were recruited from the rheumatology clinics at Addenbrooke's Hospital, Cambridge, United Kingdom. Patients with manifest CVD, untreated hypertension (blood pressure ≥140/90 mm Hg), diabetes, hypercholesterolemia (total cholesterol ≥6.5 mmol/L), or renal disease and current smokers were excluded because these conditions are associated with endothelial dysfunction and arterial stiffening.^34^ To determine whether baseline endothelial function and aortic stiffness were abnormal in RA patients, 33 age‐ and sex‐matched healthy control participants were randomly selected from our database to compare baseline endothelial function and aortic stiffness. A favorable ethics opinion was obtained from the National Research Ethics Service, and written informed consent was obtained from each participant prior any study procedures.

### Pilot Proof‐of‐Concept Study

We conducted a small pilot study in 5 healthy nonsmoking control participants (mean age 27±6 years) to test whether a single dose of BH_4_ 400 mg would improve endothelial function. We found that flow‐mediated dilatation (FMD) response increased (from 5.79±1.77% to 11.37±4.05%; *P*=0.009), and there was a significant change in the baseline diameter (from 3.77±.52 to 4.10±0.62 mm; *P*=0.008); however, there was no change in mean arterial pressure (*P*=0.1) or aPWV (*P*=0.2). Data are presented as mean±SD, and significance was determined using the paired Student *t* test. Based on these pilot data, the 400 mg once‐daily dose was deemed sufficient for the main study in RA patients and was similar to doses used previously in other conditions.^19^, ^33^

### Experimental Protocol

The present study was conducted in a randomized, double‐blind, crossover manner. To determine whether acute changes were sustained, patients were allocated to 1 of 2 groups: acute or short‐term BH_4_ supplementation.

**Figure 1:**
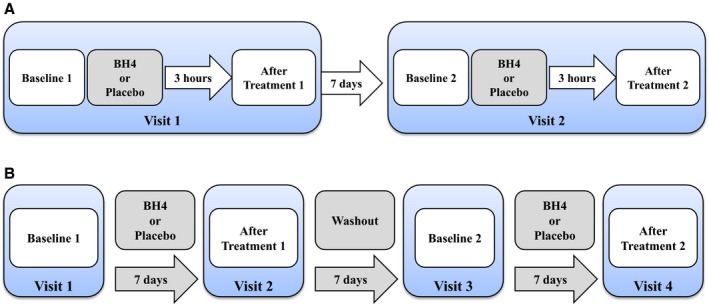
Schema of the study design. A, All participants made 2 visits, separated by 1 week. At each time point, blood pressure, arterial stiffness, and endothelial function were assessed, and a blood sample was taken. B, All participants made 4 visits to the unit on days 0, 7, 14, and 21. At each visit, blood pressure, arterial stiffness, endothelial function, and disease activity were assessed, and a blood sample was taken. BH_4_ indicates tetrahydrobiopterin.

In the acute study, participants made two 6‐hour visits to the vascular laboratory (Figure 1A). On day 1, following the baseline measurement of endothelial function, aortic stiffness, DAS28, and venous blood sampling, participants received a single oral dose of BH_4_ 400 mg or placebo (random assignment). At 3 hours after the administration of BH_4_ or placebo, the hemodynamic measurements were repeated. A 3‐hour time point was chosen based on published pharmacokinetic data.^35^ One week later, the same protocol was followed, and participants again received either BH_4_ or placebo.

In the short‐term supplementation study, participants made 4 visits, each separated by 1 week. Participants received BH_4_ 400 mg once daily or placebo (random order), each for 1 week, with 1‐week washout between treatments (Figure 1B). All hemodynamic measurements were assessed at baseline and at the end of each 7‐day treatment period (treatment 1, washout, and treatment 2). Blood was drawn at each time point for the measurement of biochemical markers, and DAS28 was calculated.

### BH_4_ Tablets

BH_4_ was given in a form of sapropterin dihydrochloride (6R‐BH_4_). 6R‐BH_4_ is the synthetic form of naturally occurring BH_4_ (BioMarin Pharmaceutical Inc) and is approved for treatment of BH_4_‐responsive phenylketonuria.

### Hemodynamic Measurements

All studies were conducted in a quiet, temperature‐controlled room. Blood pressure was recorded in the brachial artery using a validated oscillometric technique (HEM‐705CP; Omron Corp). Radial artery waveforms were obtained with a high‐fidelity micromanometer (SPC‐301; Millar Instruments) from the wrist, and a corresponding central waveform was generated using a validated transfer function (Sphygmocor; AtCor Medical). Augmentation index (AIx), a composite measure of wave reflection, mean arterial pressure and heart rate were determined using the integrated software. Aortic and brachial pulse wave velocities were measured, as previously described.^36^

Endothelial function was assessed in the brachial artery using the FMD technique.^37^ Vessel diameter was measured using high‐resolution vascular ultrasound (Acuson Aspen; Siemens AG) with a 10.0‐MHz linear‐array transducer. Brachial artery diameter was measured continuously for 1 minute at baseline and for a further 5 minutes following cuff deflation. The cuff was placed below (distal to) the ultrasound transducer and inflated to 200 mm Hg for 5 minutes. After return to baseline, vessel diameter was again measured continuously for 5 minutes following administration of 25 μg of sublingual glyceryl trinitrate. FMD was defined as the maximum percentage increase in vessel diameter during reactive hyperemia; glyceryl trinitrate–mediated dilatation was defined as the maximum percentage increase in vessel diameter after sublingual glyceryl trinitrate. FMD recordings were analyzed offline by a blinded operator using Cardiovascular Suite Software (Quipu).

### Laboratory Measurements and DAS28

Fasting lipid profile, high‐sensitivity C‐reactive protein (CRP), and erythrocyte sedimentation rate (ESR) were determined using standard methodology at the Biochemistry Laboratory at Addenbrooke's Hospital.

DAS28 is a validated composite disease activity score. The components of DAS28 include the number of swollen and tender joints from 28 assessed joints, ESR, and patient‐assessed visual analog score of overall well‐being (scale 0–100). DAS28 was calculated as described previously.^38^

### Data Analysis

The primary outcome of the study was the difference in FMD response between BH_4_ and placebo treatments. Secondary outcomes were differences in aPWV between treatments, the relationship between changes in aPWV and FMD, and the relationship between CRP and disease activity and change in FMD and aPWV.

Based on our pilot data and published data on BH_4_ supplementation, we calculated that a total of 15 patients (per study) were needed to enter the 2‐treatment crossover study to provide >80% power at *P*<0.05 to detect a 4.0‐U difference in FMD response between treatments with an SD of 5.0. Data were analyzed using SPSS software (version 21.0; IBM Corp). Two‐way repeated‐measures ANOVA was used to investigate the effect of the treatment. Custom hypothesis testing (simple) of within‐participant contrasts was performed for comparison of BH_4_ versus placebo, in which treatment order was entered as a between‐participant factor to investigate whether the order in which BH_4_ and placebo were received influenced the outcome. In post hoc tests, the effect of individual treatments was determined using paired Student *t* tests with Bonferroni adjustment for 2 comparisons. In addition, in post hoc tests, the use of methotrexate (MTX) was entered as a between‐participant factor to establish whether dyhydrofolate reductase inhibitor use influenced the BH_4_ response. For the skewed variables (CRP and ESR), log‐transformed values were used for the analyses. Differences between the RA and control groups were determined using the unpaired Student *t* test. A probability of <0.05 was considered significant. Data are given as mean±SD.

The authors had full access to the data and take full responsibility for its integrity. All authors have read and agreed to the manuscript as written.

## Results

Thirty‐three patients with active RA (mean DAS28 4.21±1.06) were studied before and after oral BH_4_ and placebo. The mean age of the participants was 57±12 years, and 27 were female. Overall, 18 and 15 participants concluded the acute and short‐term supplementation studies, respectively. There were no differences in age, BMI, DAS28, CRP, ESR, or blood pressure between groups; however, total cholesterol was slightly higher in the participants allocated to the short‐term treatment group (4.4±0.9 versus 5.4±1.1 mmol/L; *P*=0.01). Baseline characteristics are detailed by group in Table 1.

**Table 1:** Baseline Characteristics and Demographics

	Acute	Short Term	Controls	*P* Value^a^
n	18	15	33	
Sex, female/male, n/n	14/4	13/2	26/7	0.5
Age, y	55±13	59±11	56±11	0.3
RA years	12±10	14±9	—	0.7
Weight, kg	71.4±14.3	70.9±10.1	71.8±16.2	0.9
BH_4_ dose, mg/kg	5.8±1.1	5.8±0.8	—	0.9
BMI, kg/m^2^	25.8±4.5	26.7±4.0	26.2±5.2	0.6
Systolic BP, mm Hg	126±17	125±13	121±9	0.7
Diastolic BP, mm Hg	73±9	77±9	77±7	0.3
MAP, mm Hg	92±12	94±11	91±7	0.5
Heart rate, bpm	74±12	68±12	66±9^b^	0.1
aPWV, m/s	7.87±2.24	7.79±1.15	6.8±1.03^b^	0.9
Augmentation index, %	26±12	31±12	23±12	0.2
Brachial diameter, mm	3.98±0.72	3.91±0.51	3.62±0.61^b^	0.8
FMD, %	3.17±2.68	3.67±1.92	6.16±3.63^b^	0.6
GTN, %	9.39±4.38	7.64±3..04	9.42±5.41	0.4
DAS28	4.34±0.92	4.05±1.22	—	0.5
CRP, mg/L	6.50±5.90	4.80±4.07	—	0.3
ESR, mm/h	25±25	16±14	—	0.2
TC, mmol/L	4.4±0.9	5.4±1.1	5.2±1.0	0.01
Statin, n	5	5	0	0.7
Anti–TNF‐α, n	5	6	0	0.5
Methotrexate, n	8	9	0	0.4
Other DMARDs, n	9	6	0	0.6
NSAIDs, n	9	8	0	0.6
Prednisolone, n (mg/day)	7 (4.7±1.5)	2 (7.50±3.5)	0	0.1 (0.1)

Values represent mean±SD. Significance was determined using an unpaired Student *t* test. BMI indicates body mass index; BP, blood pressure; bpm, beats per minute; CRP, C‐reactive protein; DAS28, disease activity score; DMARDs, disease‐modifying antirheumatic drugs; ESR, erythrocyte sedimentation rate; NSAIDs, nonsteroidal anti‐inflammatory drugs; TC, total cholesterol; TNF‐α, tumor necrosis factor α.

a *P* value for the comparison between acute and short‐term study participants.

b *P*<0.05 between RA patients and control participants.

### Acute BH_4_ Supplementation

**Figure 2:**
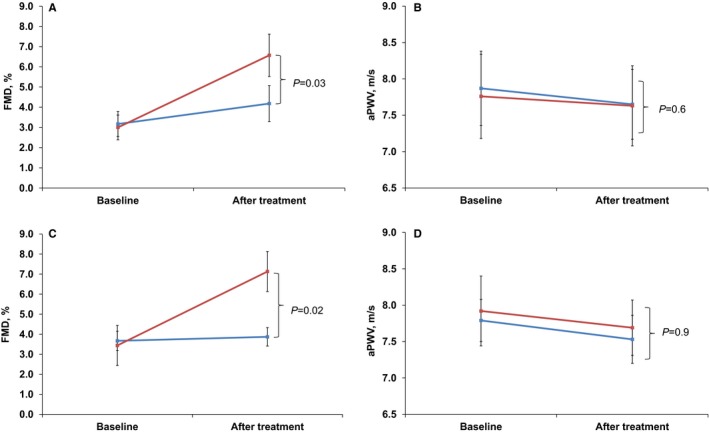
The effect of BH_4_ 400 mg. Data represent means and SEM. Significance was determined using 2‐way repeated‐measures ANOVA and Bonferroni‐corrected post hoc tests. Red represents BH_4_; blue represents placebo. A, Endothelial function was measured before and 3 hours after BH_4_ and placebo in random order. ANOVA:* P*=0.03 between groups; post hoc BH_4_: *P*=0.015; placebo: *P*=0.5. B, Aortic stiffness was measured before and 3 hours after BH_4_ and placebo in random order. ANOVA between groups: *P*=0.6; post hoc BH_4_: *P*=0.5; placebo: *P*=0.6. C, Endothelial function was measured before and 7 days after BH_4_ and placebo in random order. ANOVA between groups: *P*=0.02; post hoc BH_4_: *P*=0.002; placebo: *P*=0.4. D, Aortic stiffness was measured before and 7 days after BH_4_ and placebo in random order. ANOVA between groups: *P*=0.9; post hoc BH_4_: *P*=0.5; placebo: *P*=0.5. aPWV indicates aortic pulse wave velocity; BH_4_, tetrahydrobiopterin; FMD, flow‐mediated dilatation.

The effect of BH_4_ supplementation on hemodynamics and vascular function are detailed in Table 2. Following a single dose of BH_4_, there was a significant increase in the FMD response, whereas placebo had no effect (+3.57±4.14% versus +1.01±3.12%, between treatments; *P*=0.03) (Figure 2A). The order in which the treatments were given did not affect the outcome (*P*=0.8). Baseline diameter of brachial artery or response to sublingual glyceryl trinitrate did not change following the treatment. There were no significant differences between the changes in blood pressure, heart rate, AIx, brachial pulse wave velocity, or aPWV (Figure 2B) following BH_4_ or placebo.

**Table 2:** Change in Disease Activity, Inflammation, and Hemodynamic Parameters Following BH_4_

	Acute			Short Term		
	BH_4_	Placebo	ANOVA *P* Value	BH_4_	Placebo	ANOVA *P* Value
DAS28				0.02±0.46	−0.09±.43	0.4
CRP, mg/L				−0.79±2.6	−0.93±3.17	0.9
ESR, mm/h				0±7	1±5	0.6
SBP, mm Hg	−2±13	−6±10	0.1	−4±11	−4±8	0.9
DBP, mm Hg	−3±7	−2±6	0.6	0±5	−1±7	0.6
MAP, mm Hg	−3±9	−5±8	0.2	−2±7	−3±7	0.8
HR, bpm	−6±7	−6±7	0.8	0±8	0±7	0.8
AIx, %	−1±7	0±6	0.3	−2±7	−1±5	0.9
bPWV, m/s	−0.14±0.74	0.09±0.77	0.6	0.07±1.14	−0.25±0.52	0.6
aPWV, m/s	−0.13±0.58	−0.22±0.43	0.6	−0.22±1.33	−0.25±0.51	0.9
Adjusted aPWV, m/s	0.15±0.82	0.31±0.80	0.4	−0.22±1.27	0.28±0.72	0.3
Brachial diameter, mm	0.14±0.37	−0.02±0.40	0.2	−0.17±0.40	0.04±0.39	0.3
FMD, %	3.57±4.14^a^	1.01±3.12	0.03	3.69±4.90^b^	0.19±2.51	0.02
GTN, %	−2.8±5.1	−1.8±2.7	0.8	0.36±2.02	1.33±2.07	0.3

Values represent mean change±SD. Significance was determined using 2‐way repeated‐measures ANOVA. The effect of individual treatments was determined in post hoc tests with Bonferroni correction for 2 comparisons when overall significance in ANOVA was *P*<0.05. Results of these comparisons are indicated by asterisks. aPWV indicates aortic pulse wave velocity; bpm, beats per minute; bPWV, brachial pulse wave velocity; CRP, C‐reactive protein; DAS28, disease activity score; DBP, diastolic blood pressure; ESR, erythrocyte sedimentation rate; FMD, flow‐mediated dilatation; GTN, glyceryl trinitrate; HR, heart rate; MAP, mean arterial pressure; SBP, systolic blood pressure.

a *P*=0.015.

b *P*=0.002.

### Short‐Term BH_4_ Supplementation

The 1‐week treatment with BH_4_ led to a significant increase in FMD compared with placebo (+3.69±4.90% versus +0.19±2.51%, between treatments; *P*=0.02) (Figure 2C). The order in which the treatments were given did not affect the outcome (*P*=0.7). Again, there was no change in blood pressure or aPWV (Figure 2D).

Seventeen of the 33 RA patients were receiving MTX, a dyhydrofolate reductase inhibitor, at the time of the study. Post hoc testing demonstrated that MTX had no impact on the effect of BH_4_ on endothelial function (*P*=0.34) for the drug×visit×MTX interaction. If anything, there was a nonsignificant trend demonstrating that those on MTX had greater improvement of FMD following BH_4_ in comparison to those not on MTX (+4.6±5.0% versus +2.49±3.40%; *P*=0.18,). This was despite no difference in baseline FMD (*P*=0.9) or brachial artery diameter (*P*=0.98).

### The Effect of BH_4_ on Inflammatory Markers and Disease Activity

Following short‐term supplementation with BH_4_ and placebo, there were no changes in CRP (−0.79±2.6 versus −0.93±3.17 mg/L; *P*=0.9), ESR (0±7 versus 1±5 mm/h; *P*=0.6), or DAS28 (0.02±0.46 versus −0.09±0.43; *P*=0.4) (Table 2.

There was no correlation between the change in FMD and change in aPWV following BH_4_ (*R*=0.27; *P*=0.20); however, the change in FMD after BH_4_ correlated negatively with age (*R*=−0.44; *P*=0.01). Baseline aPWV was significantly (*P*<0.05) correlated with mean arterial pressure (*R*=0.52), age (*R*=0.52), CRP (*R*=0.39), and ESR (*R*=0.66).

### Baseline Comparison With Healthy Controls

The mean age of the healthy control group was 56±11 years. At baseline, RA patients had reduced endothelial function in comparison to control participants (FMD 3.39±2.36% versus 6.16±3.63%; *P*=0.001). After treatment with BH_4_, the FMD response was no longer different between RA patients and controls (6.82±4.83% versus 6.16±3.63%; *P*=0.5). Baseline aPWV was higher in the RA group compared with controls (7.83±1.80 versus 6.8±1.03 m/s; *P*=0.006), whereas blood pressure did not differ between the groups (Table 1).

## Discussion

The main findings of the present study are that both acute and short‐term therapy with BH_4_ significantly improves endothelial function, as assessed by vasodilatory response to reactive hyperemia in patients with active RA, but does not affect blood pressure, AIx, aPWV, or brachial pulse wave velocity. These findings suggest that modulation of endothelial function does not ameliorate arterial stiffness in RA.

The current study demonstrates, for the first time, that oral BH_4_ supplementation improves endothelial function in patients with RA by ≈3.5%, restoring it to levels comparable to those of healthy controls. The improvement of FMD was inversely associated with age, suggesting that endothelial dysfunction in younger RA patients may be more reversible than in older patients. FMD has been validated recently as a surrogate marker of future cardiovascular risk. Yeboah et al demonstrated that FMD is a predictor of incident cardiovascular events and that it improves the classification of participants as low, intermediate, and high CVD risk compared with the Framingham risk score in a large population‐based cohort.^4^ The mean FMD in their whole cohort was 4.4±2.8% versus 3.4±2.5% in incident cases, suggesting that the improvement seen in our study is likely to have a clinical impact on cardiovascular risk reduction in patients with RA.

Previous studies have demonstrated that intra‐arterial administration of BH_4_ improves endothelium‐dependent dilatation in patients with hypercholesterolemia,^27^ coronary artery disease,^28^ and type II diabetes.^29^ Oral BH_4_ supplementation also improves endothelial function in elderly patients,^30^ in long‐term smokers,^31^ and in patients with hypercholesterolemia^32^ and hypertension.^33^ Conversely, Cunnington et al showed that although oral BH_4_ led to an ≈2.5‐fold increase of plasma BH_4_ levels in patients with established coronary artery disease, it had no effect on vascular function or superoxide production because of systemic oxidation of BH_4_ to BH_2_.^19^ The impact of BH_4_ on endothelial function has not been assessed previously in RA; however, other interventions (eg, individually designed exercise program) have been shown to improve endothelial function in RA patients.^39^ Because intracellular BH_4_ levels are regulated by de novo synthesis of BH_4_ from guanosine triphosphate and through recycling of BH_2_ to BH_4_ via dyhydrofolate reductase,^40^ use of dyhydrofolate reductase inhibitors (eg, disease‐modifying antirheumatic drugs, MTX) may inhibit the bioavailability of BH_4_ in the endothelium. Nevertheless, we found no difference in the effect of BH_4_ supplementation on endothelial function between those RA patients on MTX and those not receiving MTX. A trend suggested that those patients on MTX had a greater increase in FMD following BH_4_ supplementation, perhaps because of reduced levels of inflammation. Notably, our study was not powered to look at this difference.

We and others have demonstrated that patients with RA have increased aortic stiffening in comparison to healthy participants,^10^, ^14^, ^15^, ^16^ and that can be reversed with immunomodulatory therapy.^10^, ^12^, ^41^ Our previous studies suggested a link between arterial stiffness and endothelial function,^17^, ^18^ but it is unclear if this relationship is causal or if they occur in parallel in response to common drivers such as increased inflammation. Interestingly, in the present study, neither a single dose nor short‐term supplementation with oral BH_4_ led to a change in aPWV, brachial pulse wave velocity, AIx, or blood pressure in comparison to placebo. Park et al recently demonstrated in patients with chronic kidney disease that 6R‐BH_4_ significantly improved AIx^42^; however, there was also a drop in blood pressure, and it appears that the change in AIx was not corrected for change in mean arterial pressure, only for heart rate. Similarly, Porkert et al showed a 15‐mm Hg reduction in systolic blood pressure following 8‐week treatment with BH_4_ 400 mg in hypertensive patients, but this study was not placebo controlled.^33^ In healthy older men, BH_4_ was shown to reduce carotid artery compliance, but there was a confounding fall in systolic blood pressure.^43^ In postmenopausal women, however, Moreau et al showed an improvement in carotid artery compliance and FMD response following a single dose of oral BH_4_ without change in mean arterial pressure.^44^

Our study also provided evidence about causality for endothelial function and aortic stiffness. We demonstrated previously that under resting conditions, local NO production modulates distensibility of muscular iliac artery in humans and that exogenous NO increases arterial distensibility^18^; however, data relating to elastic aorta are more controversial. A recent study by Butlin et al showed that exogenous NO donor sodium nitroprusside does not alter aortic stiffness in vivo in rats.^45^ Nevertheless, numerous studies,^10^, ^11^, ^46^ including ours, have demonstrated a reduction in aortic stiffness, which mirrors the changes in endothelial function, following anti‐inflammatory or cholesterol‐lowering therapies. The current study clearly demonstrated that improvement of endothelial function, without change in inflammation or lipids, does not lead to reduction of aortic stiffness, strongly suggesting that there is no direct causal link between aortic stiffness and endothelial function. We cannot exclude the possibility that this finding is specific to RA because previous studies have demonstrated changes in aPWV in healthy participants in acute models of induced inflammation.^47^, ^48^

### Limitations

We did not assess serum BH_4_ and BH_2_ levels in our study; however, numerous studies have demonstrated that supplementation with 6R‐BH_4_ leads to increase in serum BH_4_ levels.^19^, ^32^ Moreover, we chose the time points based on published findings. Fiege et al demonstrated that the maximal plasma biopterin levels peaked between 1 and 4 hours, with median peak of 3 hours and maximum concentration of 258.7 to 295.0 nmol/L for 10 mg/kg; even 24 hours after administration, 6R‐BH_4_ levels were still 4–5 times the basal values.^35^ Moreover, our treatment period of 1‐week was very short, and we can neither extrapolate these results to what may be the chronic effects of BH_4_ supplementation in patients with RA nor predict whether it may have beneficial effects on future cardiovascular events.

## Conclusion

This study shows for the first time in patients with RA that oral BH_4_ improves endothelial function as measured by a response to reactive hyperemia. These findings suggest that recoupling of eNOS using cofactor BH_4_ may provide a rational therapeutic approach to prevent CVD in patients with chronic inflammatory diseases. BH_4_ supplementation did not appear to reduce aortic stiffness, suggesting that endothelial dysfunction is not a pathophysiology behind aortic stiffening in RA and that these conditions may just exist in parallel, both influenced by common risk factors such as inflammation and increased reactive oxygen species production.

## Sources of Funding

Mäki‐Petäjä and Wilkinson were funded by British Heart Foundation. Wilkinson, Cheriyan and Shenker received funding from the Comprehensive Local Research Network and Wilkinson and Cheriyan from the National Institute for Health Research: Cambridge Biomedical Research Centre.

## Disclosures

None.
